# Individual quality of life in patients with multiple myeloma

**DOI:** 10.1186/2193-1801-2-397

**Published:** 2013-08-23

**Authors:** Julia Dürner, Hans Reinecker, Herbert Csef

**Affiliations:** Department of Psychosomatic Medicine and Psychotherapy, Clinic of Internal Medicine II, Julius-Maximilians-University of Würzburg, Würzburg, Germany; Department of Clinical Psychology and Psychotherapy, Otto-Friedrich-University of Bamberg, Bamberg, Germany

**Keywords:** Cancer, Psychooncology, Individual quality of life, Multiple myeloma

## Abstract

**Objective:**

The situation of patients with multiple myeloma, whose treatment often implies high-dose chemotherapy and stem cell transplantation that can be associated with severe symptoms and psychological distress, has gained attention in recent psychooncological research. This study followed an idiographic approach in order to identify the areas of life most relevant for the interviewed myeloma patients’ quality of life (QoL) as well as their current satisfaction with these.

**Methods:**

64 patients took part in semi-structured interviews according to the SEIQoL-DW Manual (Schedule for the Evaluation of Individual Quality of Life – Direct Weighting). Visual analogue scales (VAS) were used to gain additional information about a general assessment of the present QoL. Qualitative data evaluation preceded quantitative processing. Groups were compared according to the time elapsed since diagnosis regarding specified areas of life, satisfaction with these and their relative weighting. SEIQoL-DW-indices were correlated to the VAS to reflect on an interindividually comparable parameter.

**Results:**

Personal social relationships were mentioned significantly more often as important for QoL than health-related aspects, and in direct comparison were weighted significantly stronger. Regarding the change of areas relevant for QoL over the time elapsed since diagnosis, there was a significant difference between groups concerning the area of spirituality. Satisfaction differed significantly between groups for the field of leisure.

**Conclusion:**

The results for the interviewed patients with multiple myeloma point out the need to take into account the importance of social and individual aspects when reflecting on QoL. Similar findings have been reported for different samples. The relevance of an individualized approach is illustrated by the fact that individually named areas of life were rated comparatively strongly in their importance for the patients’ QoL. An overall assessment for the current QoL by means of VAS is regarded as an adequate supplement to the SEIQoL-Profile and an alternative to the SEIQoL-DW-Index.

## Background

Quality of life has increasingly gained importance as a standard in oncology and is also mentioned in the field of research as a relevant criterion for medical activities (Browne et al. 1997). The conceptualization of ‘health-related quality of life’ has established itself in quality of life research in medical contexts (Bullinger 1997). Respective measures frequently comprise areas of life that are considered as relevant according to the applied definition or by expert opinion. Content is dominated by items related to health, illness and symptoms, often focusing on health-related quality of life. A prioritization of health-related aspects in the evaluation of quality of life in this context is questioned by the results of research groups who interviewed cancer and ALS (amyotrophic lateral sclerosis) patients on important areas of life for their quality of life (Fegg et al. 2005; Frick et al. 2004; Waldron et al. 1999; Wettergren et al. 2008). The use of standardized questionnaires has been commented on with regards to universality and consistency of relevant areas of life, as well as to implicitly weighting them equally (Browne et al. 1994; Hickey et al. 1999; Jenkinson & McGee 1997). Individualized instruments have shown to reveal areas of life not typically included in standardized instruments but nominated as relevant by the respondents (Waldron et al. 1999; Wettergren et al. 2008). The use of individualized instruments can contribute to gaining a comprehensive picture with special attention to the views of the persons concerned and has been recommended for monitoring patient care (McHorney & Tarlov 1995; Wettergren et al. 2011; Molassiotis et al. 2011; Hickey et al. 1996). The situation of patients with multiple myeloma, whose treatment often implies high-dose chemotherapy and stem cell transplantation that can be associated with severe symptoms and psychological distress, has gained attention in recent psychooncological research (Frick et al. 2004; Wettergren et al. 2008; Molassiotis et al. 2011).

In the present study, semi-structured interviews were used following an idiographic approach. The objective was to explore the perspective of patients with a specific haematological malignancy and the impact of treatment on the assessment of quality of life by means of identifying areas of life relevant for the interviewed myeloma patients’ quality of life, their current satisfaction with these and their relative weighting. Personal social relationships were hypothesized to be most important in the individual assessments of quality of life. The answers of patients during the first year after diagnosis and of patients whose diagnosis dated back more than one year were compared regarding the above-mentioned aspects.

## Methods

### Participants and procedure

The study procedure was previously approved by the Medical Faculty’s Ethics Committee of Würzburg University. The data were collected in the Clinic for Internal Medicine II of Würzburg University from May to December 2011. All but six of the approached 70 persons volunteered to take part in an interview. Written informed consent was obtained from all study participants. All interviews were administered by the first author, either in the patient’s room, an examination room on the ward, or in an office, according to the participant’s preference and physical condition.

### Measures

Data collection followed the SEIQoL-DW Manual (Hickey et al. 1996; O'Boyle et al. 1993). This semi-structured interview has been used to evaluate individual quality of life in various samples, e. g. healthy individuals (Browne et al. 1994) as well as patients with congenital heart disease (Moons et al. 2005), stroke survivors (LeVasseur et al. 2005), patients with ALS (LoCoco et al. 2005), and patients with advanced cancer (Waldron et al. 1999). Several research groups have reported data on psychometric properties of the SEIQoL-DW with good results for validity, reliability and sensitivity (Waldron et al. 1999; Patel et al. 2003; Moons et al. 2004; Neudert et al. 2001). During the administration, participants are asked to name the five areas of life currently most important for their quality of life. They then rate their satisfaction with these areas during the last seven days ranging form 0 to 100. Lastly, the relative importance of each area is determined with a dynamic pie chart which allows to try out different combinations. According to the manual, an index can be calculated by multiplying current satisfaction and relative weighting of each area and summing up the results. Additionally, visual analogue scales were used to gain information about a general assessment of the present quality of life (Moons et al. 2004). The last seven days were defined as a reference period in the instructions for the SEIQoL-DW as well as for the visual analogue scales.

### Data analyis

The interview data material was screened and then categorized. Inductive formation of categories followed Mayring’s description of the procedure of summarizing qualitative content analysis (Mayring 2008). The category system was reviewed and adapted in a continuous process. The objective was to structure the obtained data for quantitative data processing in order to test hypotheses, but to develop the categories close to the original data so that individually relevant areas for quality of life assessment could be displayed. The categories were defined to comprise mentions of the generic term as well as subordinate terms and their descriptions. To test hypotheses, the named areas of life were further condensed to main categories; e. g. ‘family’ and ‘friends’ were both allocated to “personal social relationships”. Cohen’s Kappa (K) was determined to evaluate category definitions. 15% of the data were selected at random and were attributed to the categories according to the category system by a person not involved in the study.

McNemar test was used to analyse whether the proportion of participants who had named at least one area of the main category “personal social relationships” was significantly larger than the rate of those who had named at least one aspect of “health” (“personal social relationships” and “health” being the two main categories that most named areas of life were assigned to). To test for differences in the relative weighting of the named areas of life with Wilcoxon signed rank test for paired samples, the data of the 46 participants who had named areas of the main categories both “personal social relationships” and “health” were used. (If a participant had named several areas of life allocated to the same main category, their relative weights were summed up and taken into account for further calculation).

Two groups were formed according to the time elapsed since diagnosis (in months) in order to compare the groups’ results as to specified areas of life, satisfaction with these and their relative weighting. To test for significant differences, Fisher’s exact test and Mann–Whitney-U-Test were used. SEIQoL-DW-indices were correlated to the visual analogue scalings to reflect on an interindividually comparable parameter. Kendall’s Tau correlation coefficient was calculated to test the correlation of SEIQoL-DW-indices and the visual analogue scalings. Findings were considered to be statistically significant for values below α = 0.05. Statistical tests were performed using the SPSS computer program, version 19.

## Results

### Participants’ characteristics

64 patients with multiple myeloma (27 women and 37 men) volunteered to take part in the study. 30 of them were in their first year of treatment after diagnosis, and 34 at a later point in time in treatment (ranging from 13 to 147 months of treatment). The median age was 60 years (range: 33–84). The participants were mainly inpatients; four of them received outpatient treatment. Further sociodemographic and clinical characteristics of the sample are presented in Table 1. No significant differences were found between the patients in the first year after diagnosis and the patients whose diagnosis dated back more than a year regarding the distribution of the characteristics gender (Chi^2^: .03, p = .862), age (t (62) = −.7, p = .488), family status (Chi^2^: 3.8, p = .862), educational level (Chi^2^: 6.92, p = .075), and active faith (Chi^2^: 1.36, p = .244). The patients received chemotherapeutic treatment, which for 52% of the sample involved high-dose chemotherapy followed by autologous stem cell transplantation.

**Table 1:** **Sample characteristics**

	n (%)	Median (range)
Total	64 (100)	
Age in years		60 (33-84)
Sex		
male	37 (57.8)	
female	27 (42.2)	
Education level		
Secondary school	37 (57.8)	
A-Level/High school degree	5 (7.8)	
University degree	22 (34.4)	
Family status		
married	48 (75.0)	
widowed	3 (4.7)	
divorced	8 (12.5)	
single	5 (7.8)	
Religious denomination		
catholic	28 (43.8)	
protestant	25 (39.1)	
none	11 (17.2)	
Active faith		
yes	54 (84.4)	
no	10 (15.6)	
Months since diagnosis		15.5 (0-147)
Time of treatment in months		11.0 (0-147)
Current disease status		
complete remission	4 (6.2)	
partial remission	18 (28.1)	
stable disease	2 (3.1)	
progressive disease	8 (12.5)	
recurrence	2 (3.1)	
beginning of treatment, no information	8 (12.5)	
no information during ongoing treatment	22 (34.4)	
Autologous stem cell transplantation(s)	33 (51.6)	
Allogenic stem cell transplantation	8 (12.5)	

### Quality of life

Table 2 presents the main categories formed from the named areas of life most important for the current quality of life and the respective frequencies. The agreement between two raters on the main categories had been evaluated for a randomly selected part of the data at = .9. Personal social relationships were named by most participants as important for their quality of life, and they were named significantly more often (p = .006, one-tailed) than health-related or other aspects. Table 3 displays the average relative weights assigned to the main categories. The 46 participants that had named both personal social relationships and health-related aspects as most important for their quality of life had weighted personal social relationships significantly stronger (p = .006, one-tailed) than health-related aspects.

**Table 2:** **Description of the main categories and respective frequencies**

Nr.	Main category	Description (including the inductively formed first categories)	Absolute frequency	Percent^a^
1)	“Personal social relationships”	Personally relevant social contacts, including the categories ‘family‘, ‘spouse‘, ‘children‘, ‘grandchildren‘, ‘friends‘	94 **	29.4
2)	“Health”	Terms or descriptions related to physical and psychological state; including the categories ‘absence of pain and other symptoms‘, ‘mobility‘, ‘positive psychological state‘, ‘physical fitness‘	69	21.6
3)	“Leisure”	Leisure activities like “hiking”, “hunt”; including the categories ‘sport‘, ‘music‘, ‘cultural activities‘, ‘travelling‘	36	11.3
4)	“Independence”	Terms and descriptions for independence/self-determination/autonomy/self-reliance	19	5.9
5)	“Financial situation”	Terms and descriptions regarding the personal financial situation, financial security, financial independence etc.	17	5.3
6)	“Spirituality”	Terms and descriptions related to faith, religion, religious practice	17	5.3
7)	“Work”	Also: “job” or descriptions of the professional activity, as well as naming the own business	16	5.0
8)	“Home”	Also: “feeling comfortable at home”, “my home”, descriptions of home and of positive associations	13	4.1
9)	“Relation to nature”	Descriptions of affinity with nature, enjoying closeness to nature, including the category ‘garden‘	10	3.1
10)	“Other”	Once or singularly (less than four times) named areas of life (not matching any of the main categories)	29	9.1

^a^The percentage refers to the total of 320 named areas of life.

** Personal social relationships were named significantly more often (p = .006, one-tailed) than health-related or other aspects.

**Table 3:** **Average relative weights for the main categories**

Rank^a^	Main category	Mean^b^	Median^b^	Minimum^c^	Maximum^c^
1	“Personal social relationships”	41.1 **	40.0	6.0	80.0
2	“Health”	29.3	23.5	2.5	80.0
3	“Other”	22.6	17.0	5.0	32.5
4	“Independence”	21.4	17.5	5.0	75.0
5	“Spirituality”	17.7	17.5	2.5	60.0
6	“Home”	15.8	16.7	5.0	34.0
7	“Leisure”	14.4	12.5	2.0	30.0
8	“Work”	13.5	13.5	5.0	25.0
9	“Financial situation”	13.2	10.0	5.0	44.0
10	“Relation to nature”	10.8	10.0	3.0	20.0

^a^According to means of relative weights.

^b^Rounded to one decimal.

^c^Relative weights of singular categories, not sum scores.

** Personal social relationships were weighted significantly stronger (p = .006, one-tailed) than health-related aspects by the 46 participants that had named both personal social relationships and health-related aspects as most important for their quality of life.

Regarding the difference between groups concerning areas relevant for quality of life of patients in the first year after diagnosis compared to patients whose diagnosis dated back more than a year (Table 4), the number of mentions for the area of spirituality differed significantly (p = .027, two-tailed; Fisher’s exact test was conducted for the main categories with the greatest differences in descriptive analysis). Mann–Whitney-U-Test showed a significant difference between groups as to satisfaction with leisure activities (p = .028, two-tailed). Due to the procedure of relative weighting (that opposes statistical independence) no inferential statistical analysis was performed. The weights assigned by the groups were only descriptively compared, with the greatest differences for “health”, “independence”, and “home”. “Independence” was in comparison weighted more strongly by participants whose diagnosis dated back more than a year, while the other two aspects were assessed as more important by participants in the first year after diagnosis than by those whose diagnosis dated back more than one year.

**Table 4:** **Comparison of group results**^**a**^

Main category	Group 1 (n = 30)			Group 2 (n = 34)		
	Patients naming at least one aspect	Satisfaction (mean^b^)	Weight (mean^b^)	Patients naming at least one aspect	Satisfaction (mean^b^)	Weight (mean^b^)
“Personal social relationships”	29 (96.7%)	80.4	37.0	31 (91.2%)	79.0	45.0
“Health”	26 (86.7%)	63.0	35.5	23 (67.6%)	62.4	22.3
“Leisure”	11 (36.7%)	35.0	11.2	16 (47.1%)	63.1 *^a^	16.6
“Independence”	11 (36.7%)	57.3	16.4	8 (23.5%)	59.4	28.3
“Financial situation”	5 (16.7%)	64.2	12.6	12 (35.2%)	68.6	13.4
“Spirituality”	12 (40.0%) *^b^	85.8	19.3	5 (14.7%)	53.0	13.7
“Work”	8 (26.7%)	53.1	12.4	8 (23.5%)	57.5	14.5
“Home”	7 (23.3%)	79.4	21.1	6 (17.6%)	80.0	9.7
“Relation to nature”	4 (13.3%)	66.3	10.1	6 (17.6%)	53.3	11.3
“Other”	8 (26.7%)	64.9	21.9	13 (38.2%)	71.9	23.8

^a^Group 1: patients during the first year after diagnosis. Group 2: patients whose diagnosis dated back more than a year.

^b^Rounded to one decimal.

*^a^Patients whose diagnosis dated back more than a year reported significantly higher satisfaction concerning leisure activities than patients during the first year after diagnosis (p = .028, two-tailed).

*^b^Aspects of the main category ”spirituality” were mentioned significantly more often by patients in the first year after diagnosis (p = .027, two-tailed).

The comparison of the values of the SEIQoL-DW-indices and visual analogue scalings showed on average higher values for the calculated indices than for the participants’ direct assessments of general quality of life (SEIQoL-DW-index mean: 71.3 and median: 71.5 vs. VAS mean: 57.7 and median: 58.0) with small differences in variability. The two parameters correlate significantly, but at a moderate level (r = .522**).

### Individual profiles

**Figures 1:**
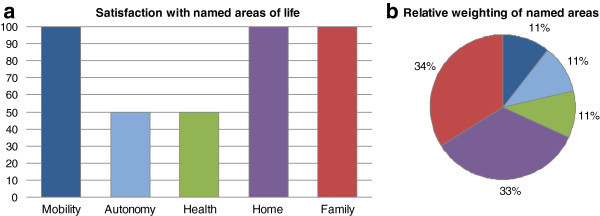
**a and b SEIQoL-DW-Profile of a 54-year-old man eleven months after diagnosis.**

**Figures 2:**
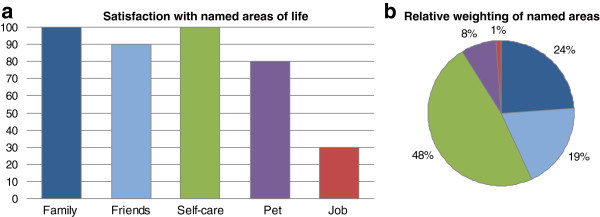
**a and b SEIQoL-DW-Profile of a 46-year old woman 77 months after diagnosis.**

Two examples of SEIQoL-DW-profiles are presented in Figures 1 and 2 to illustrate how differently quality of life can be evaluated at an individual level.

Figures 1a and 1b display the assessment given by a 54-year-old man whose multiple myeloma had been diagnosed eleven months ago. He was in treatment before a first autologous stem cell transplantation. The most important areas of life for quality of life to him were mobility (“that I am able to walk and drive the car”), autonomy (“that I can move around the flat without any help”), health, home, and the contact especially to one person of his family. He reported his view on health to have changed, and that it was much more important to him now than it used to be. He rated his current quality of life in general at 60 of 100 on the VAS, and the calculation of the index added up to 89.3.

Figures 2a and 2b summarize the results of the interview with a 46-year-old woman who had been diagnosed with a smouldering myeloma 77 months ago. At the time of the interview, she was in outpatient treatment after two autologous stem cell transplantations that had been administered after the disease progressed to a stadium that required treatment five years after the diagnosis. The participant described how her way of looking after herself had changed a lot during coping with the disease. She would now pay much more attention to what was good for her. Her job had become less important compared to former times, but she still wished to be able to work again and thus rated work among the most important areas. She assessed her general current quality of life at 86 on the VAS; the value for the index resulted in 95.8.

## Discussion

The data support the vital importance of personal social relationships and individual aspects for quality of life. The present interpretation does not imply disregarding the relevance of health-related aspects. Rather, the results point out the need to take into account the importance of social and individual aspects when reflecting on quality of life. Similar findings are reported for more heterogeneous samples of patients with different malignancies. The special meaning of social relationships for quality of life – also in a life situation of severe illness, when health-related aspects have a strong presence and everyday relevance – is underlined by several publications where relationships were the or among the most nominated areas relevant for the quality of life (Fegg et al. 2005; Wettergren et al. 2008; Wettergren et al. 2003; Taminiau-Bloem et al. 2010). These data hint to the limits of quality of life conceptions that focus too strongly on health-related aspects.

Regarding the areas relevant for quality of life, there was a significant difference between groups for the area of spirituality: Respective areas of life were mentioned significantly more often by participants in the first year after diagnosis than by those whose diagnosis dated back more than a year (a Chi^2^-test had shown no significant difference between groups for active faith and other characteristics mentioned above). This can be related to the meaning of religious and spiritual aspects during the coping process and may hint to the interpretation that they are especially important when patients initially adapt to the new situation. Administered in a specific context, repeated measurements with two samples of cancer patients treated with palliative intent reported only small numeric differences in the number of mentions for spiritual aspects between two interviews (Sharpe et al. 2005; Echteld et al. 2005). Wettergren et al. found no mentions of spirituality in their administration of the SEIQoL-DW to patients with haematological malignancies before and one year after stem cell transplantation (Wettergren et al. 2008). Gathering more data in longitudinal studies with repeated measurements in could help to clarify the role of spiritual coping, changes regarding the concept of health and other response shift processes in different stages of coping with cancer. The result of a significant difference as to satisfaction with the field of leisure can point to actual changes in recreational activities or to response shift. It is an outstanding feature of individualized measures like the SEIQoL-DW that response shift processes can be captured directly in repeated measurements.

The significance of an individualized approach is illustrated by the fact that individually named areas of life, e. g. “my pet”, “sleep”, “nutrition”, “self-care”, were rated comparatively strongly in their importance for the individual quality of life (compare Table 3). The moderate correlation between the visual analogue scaling assessments and the calculated indices illustrates the assumption that different aspects are captured by both measures; and there is obviously a difference between asking for the status and weighting of the most important areas of quality of life and asking for an overall assessment of quality of life. On average higher scores for the SEIQoL-DW-Index compared to the assessments on visual analogue scales are also reported by Frick et al. (Frick et al. 2004) and Pearcy et al. (Pearcy et al. 2008). The visual analogue scaling may be influenced by factors that are not represented in the determination of the Index. Since the majority of participants in our study were inpatients, aspects of the present situation such as treatment side effects, absence from home etc. may have affected their global quality of life-assessment in the visual analogue scales to a greater extent than their evaluation of the most important areas in the SEIQoL-DW. The SEIQoL-DW authors ask for careful interpretations of the Index and its interindividual comparability (Jenkinson & McGee 1997; O'Boyle et al. 1993), and the original SEIQoL Manual included a visual analogue assessment. An overall assessment for the current quality of life by means of visual analogue scales is regarded as an adequate supplement to the SEIQoL-Profile and an alternative to the SEIQoL-DW-Index. Rather than reducing quality of life assessments to an interindividually comparable parameter (be it the Index or via VAS), the areas of life named by the participants, satisfaction with these und their weighting should be considered when comparing assessments.

The procedure of administering the SEIQoL-DW interview was described inconsistently by different authors, asking participants either to rate their current satisfaction with the areas or the current status of functioning of the areas, and thus highlighting rather subjective or objective aspects (Wettergren et al. 2009). In this study, we decided to focus on the more subjective aspect of satisfaction considering the special relevance of subjective evaluations compared to objective factors for quality of life assessments (Waldron et al. 1999; Moons et al. 2004; Herschbach 2002). The manual instruction regarding the relative weighting of the areas was extended by one sentence labelling equal and different weights for the named areas as equivalent options in order to avoid biases.

The SEIQoL-DW-Manual’s instruction to ask for the *five* areas of life most important to the current quality of life is a limitation to the present study. This regulation was not changed when planning the study for reasons of adherence and comparability. Nevertheless, objections regarding the independence of the named areas and their weighting were considered during data analysis. The SMiLE (Schedule for Meaning in Life Evaluation) is an analogously designed measure, whose authors decided in the course of their studies to abandon asking for a fixed number of areas of life (Fegg et al. 2010) (this instruction had been introduced in the SEIQoL-Manual for methodological reasons that expired with the development of the SEIQoL-DW-Disc). In terms of consequently realising an idiographic approach, dropping this requirement should be considered for future applications of the SEIQoL-DW as well. That would also make the potentially problematic procedure of presenting a prompt list to participants who name less than five areas of life dispensable (Westerman et al. 2006). (In the present study, this was relevant for less than 4% of the data).

Setting effects must be kept in mind as most of the interviews were conducted during inpatient treatment. This context may particularly trigger feelings of fear and helplessness that can influence the reflection on and assessment of quality of life. During some of the interviews held in the patient’s room there were other persons present (11 interviews, i. e. 7%), e. g. other patients when both patients were limited in their mobility and the participant declared not to be disturbed by the presence of the other patient or visiting relative. This implies that social desirability effects cannot be excluded, also because the interviews were administered by a staff member of the hospital (not involved in patient care for the interviewed patients).

## Conclusion

The medical treatment of haematological malignancies is continually improving, and the impact on quality of life is of growing interest, especially in the context of intensive treatments such as stem cell transplantations (Montgomery et al. 2002). This study focused on an idiographic approach to explore and describe relevant areas of life for the quality of life of patients with multiple myeloma. The results underline how important it is in the clinical context to ask patients which aspects they rate most important for their quality of life, e. g. in the context of treatment related decisions. Depending on the objective, standardized and individualized measures can be regarded as complementing each other (Wettergren et al. 2008). Repeated measurements with measures that take into account not only health-related, but also social and individual aspects can provide a different perspective on the experiences of the persons concerned and can help to better understand relevant factors for quality of life in the course of treatment (Osbourne et al. 2012).
